# Identification of Inhibitors Targeting Ferredoxin-NADP^+^ Reductase from the *Xanthomonas citri* subsp. *citri* Phytopathogenic Bacteria

**DOI:** 10.3390/molecules23010029

**Published:** 2017-12-24

**Authors:** Marta Martínez-Júlvez, Guillermina Goñi, Daniel Pérez-Amigot, Rubén Laplaza, Irina Alexandra Ionescu, Silvana Petrocelli, María Laura Tondo, Javier Sancho, Elena G. Orellano, Milagros Medina

**Affiliations:** 1 Departamento de Bioquímica y Biología Molecular y Celular, Facultad de Ciencias, and Institute of Biocomputation and Physics of Complex Systems (BIFI-IQFR and CBsC-CSIC Joint Units), Universidad de Zaragoza, Pedro Cerbuna, 12, 50009 Zaragoza, Spain; mmartine@unizar.es (M.M.-J.); ggoniunizar@gmail.com (G.G.); danielperezamigot@hotmail.com (D.P.-A.); elru13@gmail.com (R.L.); Iri.ale.ionescu@gmail.com (I.A.I.); jsancho@unizar.es (J.S.); 2 Departamento de Química Física, Universidad de Santiago de Compostela, 15782 Santiago de Compostela, Spain; 3 Molecular Biology Division, Instituto de Biología Molecular y Celular de Rosario (IBR), CONICET, Facultad de Ciencias Bioquímicas y Farmacéuticas, Universidad Nacional de Rosario, Rosario 2000, Argentina; spetrocelli79@gmail.com (S.P.); tondo@ibr-conicet.gov.ar (M.L.T.); orellano@ibr-conicet.gov.ar (E.G.O.)

**Keywords:** activity-based high-throughput screening, enzyme inhibitors, ferredoxin-NADP(H) reductase, *Xanthomonas citri* subsp. *citri*

## Abstract

Ferredoxin-NADP(H) reductases (FNRs) deliver NADPH or low potential one-electron donors to redox-based metabolism in plastids and bacteria. *Xanthomonas citri* subsp. *citri* (*Xcc*) is a Gram-negative bacterium responsible for citrus canker disease that affects commercial citrus crops worldwide. The *Xcc fpr* gene encodes a bacterial type FNR (*Xcc*FPR) that contributes to the bacterial response to oxidative stress conditions, usually found during plant colonization. Therefore, *Xcc*FPR is relevant for the pathogen survival and its inhibition might represent a strategy to treat citrus canker. Because of mechanistic and structural differences from plastidic FNRs, *Xcc*FPR is also a potential antibacterial target. We have optimized an activity-based high-throughput screening (HTS) assay that identifies *Xcc*FPR inhibitors. We selected 43 hits from a chemical library and narrowed them down to the four most promising inhibitors. The antimicrobial effect of these compounds was evaluated on *Xcc* cultures, finding one with antimicrobial properties. Based on the functional groups of this compound and their geometric arrangement, we identified another three *Xcc*FPR inhibitors. Inhibition mechanisms and constants were determined for these four *Xcc*FPR inhibitors. Their specificity was also evaluated by studying their effect on the plastidic *Anabaena* PCC 7119 FNR, finding differences that can become interesting tools to discover *Xcc* antimicrobials.

## 1. Introduction

Bacteria of the *Xanthomonas* genus are aerobic Gram-negative γ-proteobacteria, some of which are phytopathogens. *Xanthomonas citri* subsp. *citri* (*Xcc*) infects most citrus plants causing the citrus canker disease, an important problem in commercial fruit agriculture [1,2]. The bacterium enters the plant through the stomata or wounds, causing necrotic lesions that reduce fruit quantity and quality, and survives in plant debris as well as in seeds, resulting in an infective cycle that is difficult to interrupt. Methods to combat citrus canker include only preventive general anti-bacterial treatments [3]. The identification of molecules able to block key metabolic processes for the bacteria’s survival and infectivity may be a feasible approach to cope with this problem.

One of the earliest responses to pathogen recognition in plant defense is the rapid generation of reactive oxygen species (ROS) at the site of infection [4]. Their accumulation in high amounts in the apoplast is called “oxidative burst” and could be considered as a specific trait during the interaction with the microorganism [5,6]. In this context, phytopathogenic bacteria must overcome an oxidative stress barrier to successfully colonize the host plant, and the bacterial enzymes involved in the bacteria oxidative stress response play relevant roles during infection. Ferredoxin-NADP^+^ reductases (FNRs, EC 1.18.1.2) are FAD dependent oxidoreductases that participate in several metabolic functions, including the bacterial response to ROS [7,8]. In *E. coli*, the *fpr* gene is one of the members of the *soxRS* regulon, and its disruption renders cells highly susceptible to the bactericidal effects of methyl viologen [9]. In addition, mutation of *fpr* and *finR* genes, the second codifying for a regulatory protein required for paraquat-dependent expression of *fpr*, results *Pseudomonas putida* cells being more sensitivity to oxidative stress [10]. Accordingly, *fpr* expression in *Xcc* was found to be induced by exposure to superoxide generating agents [11]. Thus, the ferredoxin-NADP^+^ reductase from *Xcc* (*Xcc*FPR) is one of the factors expected to contribute to the response of this pathogen to maintain infection [11]. Therefore, molecules inhibiting *Xcc*FPR would help to reduce *Xcc* scavenging of ROS, and could be applied in a synergistic manner with other tools to more efficiently attenuate the bacterial response.

**Figure 1:**
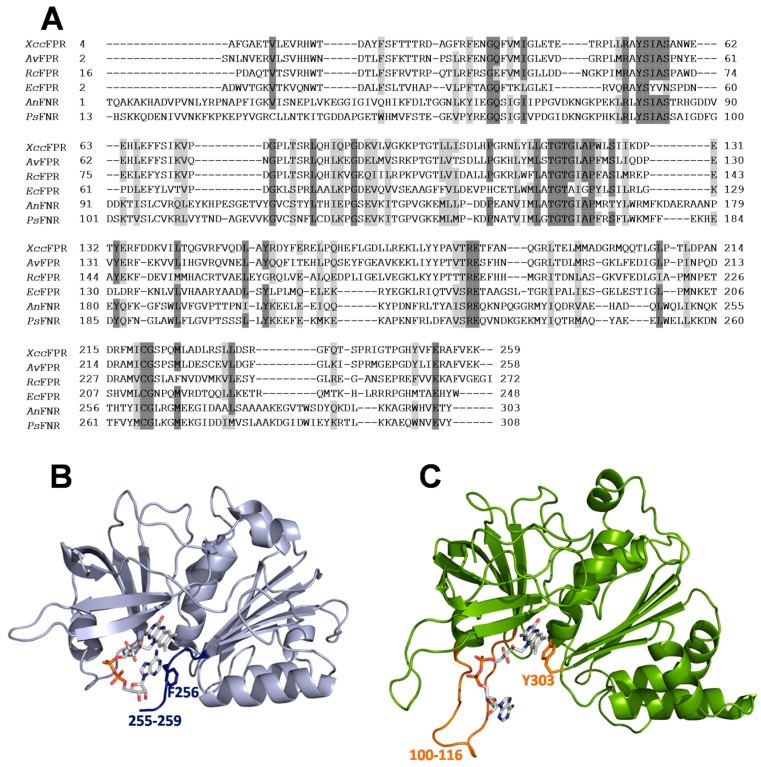
(**A**) Structural alignment of bacterial FPRs from *Xcc* (*Xcc*FPR), *Azotobacter vinelandii* (*Av*FPR), *Rhodobacter capsulatus* (*Rc*FPR) and *Escherichia coli* (*Ec*FPR) and plastidic FNRs from *Anabaena* PCC 7119 (*An*FNR) and *Pisum sativum* (*Ps*FNR). Similarities are shown in light grey and identities in dark grey. Structural models for (**B**) *Xcc*FPR (4b4d, light blue) and (**C**) *An*FNR (1que, green). Proteins are in cartoon and FAD cofactors are in sticks. In *Xcc*FPR, A255 faces the flavin *re*-face (shown in sticks) and is followed by a C-terminal extension (dark blue) that stacks on the adenine moiety of FAD contributing to its folded conformation. In *An*FNR, the residue stacking the flavin re-face is the C-terminal Y303 (orange sticks), and the FAD is in an extended conformation towards the 100-116 loop (orange) that is absent in FPRs.

Plant-type FNRs are subdivided into plastidic-type (herein FNR) and bacterial-type (herein FPR and including *Xcc*FPR). The bacterial-types catalyze the electron transfer from NADPH to different electron transfer proteins and are mainly involved in nitrogen fixation and detoxification (including ROS) processes [11,12]. On the contrary, the main function of plastidic-type FNRs is to catalyze the photosynthetic electron transfer from PSI to NADP^+^ to produce NADPH, but they are also able to efficiently provide electrons from NADPH to several electron carrier proteins [7,8,13,14,15]. Generally, the efficiency of FNRs is several orders of magnitude higher than that of FPRs. Differences in the geometric disposition of the reacting atoms in the catalytic competent complexes, together with the active sites of FPRs being more organized and rigid during catalysis, are claimed as the key factors for the dissimilar reactivity patterns [7,12,16,17]. Thus, contrary to that observed in the active site of FNRs, the residue facing the flavin isoalloxazine ring in FPRs is not the last residue of the sequence (Figure 1). In addition, a Phe or Trp residue at the C-terminal extension of FPRs stacks on the adenosine moiety of FAD contributing to its folded conformation versus the extended one presented by FAD in FNRs (Figure 1) [7,11,18,19,20]. These distinct organizations agree with differences in coenzyme approaching and geometry of reacting complexes, and suggest that the catalytically competent allocation of the nicotinamide in the active site will differ between FPRs and FNRs [7,21]. Such structural dissimilarities between FNRs and FPRs envisage the selection of molecules targeting *Xcc*FPR and having no effect on their plastidic homologous as feasible.

In this study we develop an activity based high throughput screening (HTS) method to seek for molecules inhibiting *Xcc*FPR. Thus, screening of two commercial chemical libraries allowed us to identify compounds that can likely be used as a starting point (lead compounds) for their future chemical improvement to better fit the protein target and to improve potency, selectivity and pharmacokinetics, as well as to become drug-like enough to be biologically tested. Overall, this study provides interesting tools that can be used for the discovery of phytosanitary compounds against citrus canker propagation.

## 2. Results and Discussion

### 2.1. An XccFPR Activity Based High-Throughput Screening to Discover Xcc Antimicrobials

The steady-state kinetic parameters for the *Xcc*FPR diaphorase activity were reported by Tondo and collaborators using ferricyanide as electron acceptor (*K_m_*^NADPH^ = 10.8 ± 0.5 μM and *k_cat_* = 122 ± 18 s^−1^) [11]. However, since the turnover of the diaphorase activity of plant-type FNRs is usually lower when using the 2,6-dichlorophenolindophenol (DCPIP) two electron acceptor, we considered that the *Xcc*FPR NADPH-dependent DCPIP-diaphorase activity was more adequate to set a 96-well plate activity based HTS method. Therefore, we first determined the kinetic parameters for this activity (Figure S1). As expected the resulting *K_m_*^NADPH^ value, 6.6 ± 1.1 μM, was in the range of that observed when using ferricyanide as acceptor, while turnover was five-fold slower, *k_cat_* = 22.6 ± 2.3 s^−1^ (*n* > 3, mean ± SD).

Because the compounds of the chemical libraries used in our HTS are solved in 100% DMSO, we checked the effect of this solvent on the enzyme activity. DMSO below 3% did not modify the kinetic parameters, but larger percentages decreased the maximal rate constant. Moreover, since interaction of DMSO with proteins can produce their precipitation and denaturation [22], we also evaluated its effect on *Xcc*FPR stability. A DMSO dose dependent destabilizing effect was evidenced when evaluating the influence of DMSO on the thermal midpoint temperature for flavin release (T_mFAD_) as an indicator of protein stability (Table S1). Herein DMSO concentrations were kept below 2.5% to ensure both the solubility of organic chemicals and protein folding.

**Figure 2:**
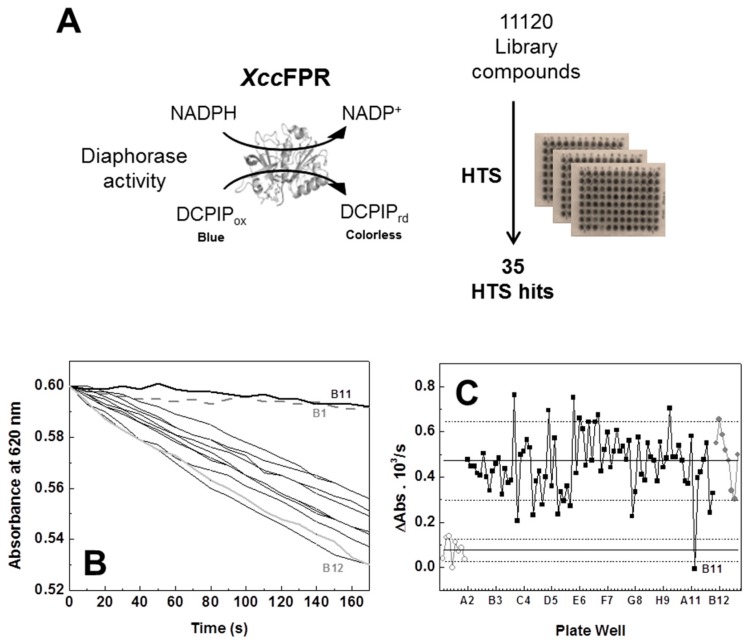
Activity-based high-throughput screening (HTS) for the discovery of the ferredoxin-NADP^+^ reductase from *Xanthomonas citri* subsp. *citri* (*Xcc*FPR) inhibitors. (**A**) Design of the activity assay and of the overall screening using as electron acceptor 2,6-dichlorophenolindophenol (DCPIP) and NADPH as electron donor. (**B**) Reaction kinetic traces for Row B of an HTS plate. Black lines correspond to wells containing library compounds (250 µM), while grey ones correspond to the negative (dashed) and positive (solid) control wells. The line in dark bold corresponds to Well B11 (behaving similar to negative controls) and this compound was selected as a potential inhibitor of *Xcc*FPR. (**C**) Rates for the *Xcc*FPR diaphorase activity (ΔAbs/s) calculated in each of the wells of one HTS plate. Data from wells containing library compounds are in black, while negative and positive controls are in grey open and grey closed circles, respectively. Solid lines represent negative and positive controls’ average velocities, and dotted lines are the average velocity plus and minus the standard deviation, respectively. The letter and number indicate the position of the well in the plate (row and column respectively) for the selected hit.

We then designed an NADPH-dependent DCPIP-diaphorase activity based HTS assay for the discovery of molecules inhibiting *Xcc*FPR using 96-well plates and the Prestwick (14 plates, 80 compounds/plate) and Maybridge (125 plates, 80 compounds/plate) libraries of compounds (Figure 2). As detailed in the Methods section, each HTS 96-well plate assay had the following: (i) in all wells of Column 1 all reactants but no enzyme or chemical from the library (negative controls); (ii) in all wells of Column 12 all reactants and enzyme (positive controls); and (iii) in wells from Columns 2–11 all the reactants, the enzyme and each of the compounds of a plate of the screening library. The reaction was followed by determining the rates for DCPIP reduction by *Xcc*FPR in each one of the wells, as indicated in the Methods section (Figure 2B shows reaction rates for the data of a row, including controls, of one of the 96-well assay plates). All wells producing reaction rates lower than 90% of the positive controls’ average activity were preselected as potentially containing compounds that can be inhibitors of the *Xcc*FPR diaphorase activity (Figure 2C shows the detail of this analysis for the 96 wells of one assay plate).

Among the 11,120 compounds of the assayed libraries, two in the Prestwick library and up to 41 in the Maybridge library reduced the positive controls’ average reaction rate more than 90%. Eight compounds out of these 43 were not commercially available in adequate quantities to continue this study, and the remaining 35 were considered as HTS hits (Table S2). At the top of these HTS hits, only C22 was identified as having properties of pan assay interference compounds (PAINS) [23].

**Figure 3:**
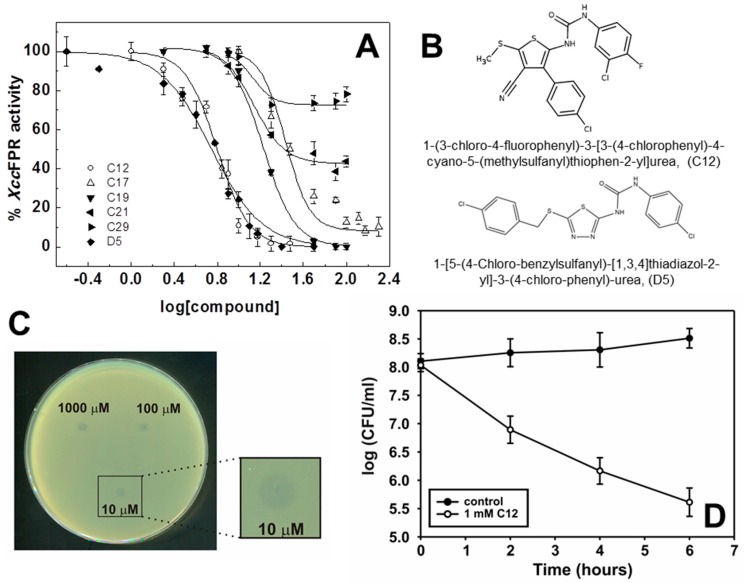
Best performing inhibitors. (**A**) Dose-response curves for the *Xcc*FPR 2,6-dichlorophenolindophenol (DCPIP)-diaphorase activity in the presence of representative hits. Values derived from these representations are included in Table 1 and Table 2. Experiments performed at 25 °C in a mixture containing 20 nM *Xcc*FPR, 95 µM DCPIP and 50 μM NADPH in 50 mM Tris/HCl pH 8.0 (*n* > 3, mean ± SD). (**B**) Chemical structures of C12 and D5. (**C**) *Xcc* growth inhibition in SB-agar plates with different concentrations (1000, 100 and 10 µM) of C12. The zones of growth inhibition were photographed after incubation for 24 h at 28 °C. (**D**) Liquid SB growth in the presence of 1 mM C12. *Xcc* culture was cultivated aerobically in SB medium at 28 °C with shaking at 200 r.p.m. Aliquots were taken at the indicated times and measured for colony-forming capacity on SB-agar plates (*n* > 3, mean ± SD). Control: 2% DMSO.

To rate the power of HTS hits as *Xcc*FPR inhibitors, we assayed them in terms of the concentration of the compound causing 50% enzyme inhibition (IC_50_) and maximal inhibition (IC_max_), as well as the percentage of remaining activity at IC_max_ (Figure 3, Table 1). 13 compounds (C2, C4, C5, C12, C15, C17, C18, C19, C21, C24, C26, C29 and C30) yielded IC_50_ values in the 7–50 µM range, but only nine of them (C2, C5, C12, C15, C17, C18, C19, C26 and C30) inhibited over 90% the enzyme activity at acceptable low IC_max_ values. Considering lower IC_50_ and IC_max_ values as well as solubility properties, we chose four HTS hits as the potentially best performing inhibitors of *Xcc*FPR; namely C5 (IC_50_ = 46 μM), C12 (IC_50_ = 7.7 μM), C17 (IC_50_ = 27 μM) and C19 (IC_50_ = 17 μM). To assess the effect of these 4 hits on *Xcc* growth, bacteria were plated on solid SB medium in the presence of increasing inhibitor concentrations. Only C12 inhibited *Xcc* growth at all concentrations assayed, the lowest being 10 µM (Figure 3C). To confirm this result, we analyzed growth inhibition by C12 in liquid medium observing a decrease of bacterial viability (Figure 3D). Given these results, we decided to continue our study using C12, 1-(3-chloro-4-fluorophenyl)-3-[3-(4-chlorophenyl)-4-cyano-5-(methylthio)-2-thienyl]urea.

To investigate the effect of the C12 functional groups, geometric arrangement and molecular scaffolds, on *Xcc*FPR inhibition, we performed a compound similarity search over the Chemical Entities of Biological Interest (ChEBI) database [24] and the Chemical Cloud Database (ChemCD, http://www.chemcd.com/) (Figure S2). We found eight potentially relevant compounds (herein named D1–D8) (Table S3) and also assessed their effect as *Xcc*FPR inhibitors (Table 2). Only D5, (1-[5-(4-chloro-benzylsulfanyl)-[1,3,4]thiadiazol-2-yl]-3-(4-chloro-phenyl)-urea), showed a similar inhibitory effect on *Xcc*FPR to C12. D2 and D8 showed moderate IC_50_ but high concentrations were required to abolish *Xcc*FPR activity, while the rest of the compounds did not show inhibitory properties. In light of this it seems that chloro-phenyl rings fused through a urea-thiadiazol cyclic ring system fulfil the structural requirements for *Xcc*FPR inhibition.

We finally assessed the selectivity of our *Xcc*FPR inhibitors by testing the diaphorase activity of the plastidic *Anabaena* PCC 7119 FNR (*An*FNR) at increasing concentrations of D2, D5 and D8 (Table 3). In terms of IC_50_ and percentage of total inhibition, D2 appears as a good inhibitor for *An*FNR as for the bacterial enzyme. IC_50_ values for D5 and D8 are above 100 µM for the plastidic enzyme, indicating that they are poorer inhibitors. High concentrations of D5 and D8 inhibit the *An*FNR activity by ~47% and ~73%, respectively. Therefore, when comparing IC_50_, IC_max_ and maximal D5 inhibition, it is envisaged that we can find D5 concentrations that considerably inhibit *Xcc*FPR but hardly affect *An*FNR (IC_50_ for *An*FNR is 6 times larger than that of *Xcc*FPR).

### 2.2. Inhibition and Binding Mechanisms of XccFPR Inhibitors

Before assessing the molecular mechanisms of the best-performing hits as *Xcc*FPR inhibitors, we needed to confirm that the observed inhibition was not due to the compounds producing the protein precipitation and denaturation. Thus, we evaluated their influence on the thermal stability of *Xcc*FPR. T_mFAD_ values determined in the presence of the selected compounds indicated that none of them had a deleterious effects on protein stability (Table S1). Moreover, they also had very mild effects as protein stabilizers, contrary to that exerted by the NADP^+^ coenzyme (Table S1).

**Figure 4:**
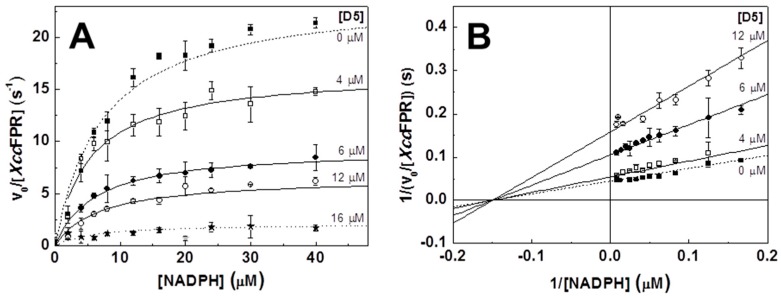
Mechanisms of the D5 *Xcc*FPR inhibitors. (**A**) Michaelis-Menten plots and (**B**) Lineaweaver-Burk representations with global fit for non-competitive inhibition. Reaction rates were measured in 50 mM Tris/HCl pH 8.0 containing 20 nM *Xcc*FPR, 95 µM DCPIP and 0–50 μM NADPH at 25 °C (*n* = 3, means ± SD). All samples contained 2% DMSO.

We then determined the inhibition mechanisms and the inhibition constants (*K_i_*) of C12, D2, D5 and D8 by measuring the *Xcc*FPR activity as a function of NADPH at increasing inhibitor concentrations. With the only exception of D2 which behaved as an uncompetitive inhibitor, the remaining compounds inhibit *Xcc*FPR in a non-competitive manner (Table 4, Figure 4). Determined *K_i_* values point to C12 and D5 as the most potent inhibitors, in agreement with their IC_50_ values.

**Figure 5:**
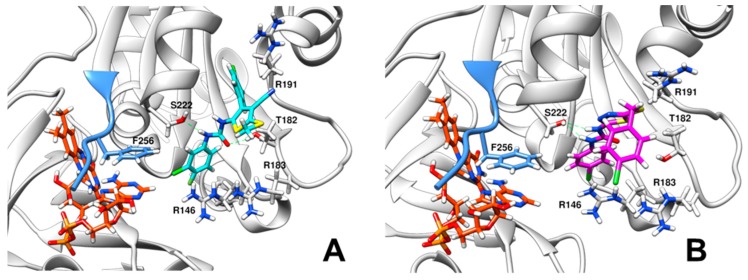
Stabilization of inhibitors in the docking binding cavities. (**A**) Detail of the C12 binding site for the best docking pose of *Xcc*FPR:C12 after 60 ps of MD simulation. (**B**) Detail of the D5 binding site for the best docking pose of *Xcc*FPR:D5 after 100 ps of MD simulation. FAD, C12 and D5 are shown in Corey-Pauling-Koltun (CPK) colored sticks with carbons in orange, light blue and pink respectively. The C-terminal extension of *Xcc*FPR is colored in blue. Side chains of docking relevant residues are highlighted as sticks. Predicted H-bonds are indicated as dashed green lines.

Since attempts to crystallize *Xcc*FPR in complexes with C12 and D5 failed, we used protein-ligand docking simulations to evaluate the possible binding modes of C12 and D5. The highest ranked docking clusters showed very similar allocations for C12 (Figure S3A), while the clusters for D5 exhibited more variability (Figure S3B). Poses for the top ranked clusters were then used as the starting point for short molecular dynamics (DM) simulations to refine protein-ligand interactions (Figure S4). The final *Xcc*FPR:C12 model points to several interactions contributing to the binding: (i) the chloro-fluorobenzene moiety of C12 laterally stacks against the π-π system formed by F256 and the adenine ring of the FAD, (ii) this benzene ring also establishes a cation-π interaction with R146, (iii) the C12 urea bridge H-bonds to S222 and T182, (iv) R183 H-bonds to the C12 thioether moiety and (v) R191 stabilizes the position of the outer chloro-fluorobenzene ring (Figure 5A). Our *Xcc*FPR:D5 model also retains the D5 urea bridge H-bonded to S222, as well as the attached chloro-benzene moiety stabilized by the F256 and R146 side-chains, and the adenine of FAD (Figure 5B). However, in this case R191 contributes to stabilizing the D5 thioether moiety and the 1,3,4-diathiazole ring rather than the D5 outer chloro-benzene moiety.

**Figure 6:**
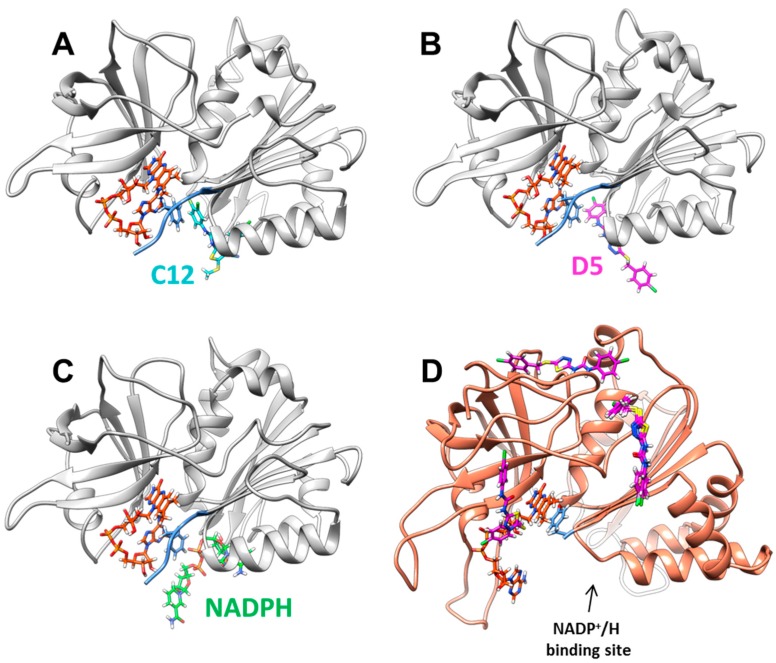
Binding pockets of selected ligands in the overall protein structure. (**A**) Model for the best docking pose of *Xcc*FPR:C12 after MD refinement. (**B**) Model for the best docking pose of *Xcc*FPR:D5 after MD refinement. (**C**) Theoretical binding of the NADPH substrate (based in PDB 4b4d for the protein and PDB 2vnj for the NADP^+^ conformation as found in *Rc*FPR). (**D**) Four best docking clusters of *An*FNR:D5. *Xcc*FPR and *An*FNR are shown as grey and light salmon cartoons respectively. FAD, C12, D5 and NADPH are shown in CPK colored sticks with carbons in orange, light blue, pink and green respectively. The C-terminal extension of *Xcc*FPR and the C-terminal residue in *An*FNR are colored in blue.

These data suggest that the chloro-phenyl-urea moiety is the key scaffold for the inhibitory activity of C12 and D5 on *Xcc*FPR. This functional group’s arrangement docks in a cavity formed at the confluence of the proposed binding site for the NADP^+^ coenzyme pyrophosphate bridge, the characteristic C-terminal extension found in FPRs (255-AFVEK-259 in *Xcc*FPR) and the adenine moiety of FAD (as a consequence of its bent conformation in FPRs) (Figure 6). Because of the C-terminus being shorter in plastidic enzymes and of their FAD showing an extended conformation that leaves its adenine moiety far from the C-terminal, such C12 and D5 binding cavity does not appear to be similarly formed in FNRs (Figure 1). In fact, the theoretical docking of C12 and D5 to *An*FNR does not find representative clusters in the NADP^+^ binding region (Figure 6D), showing them preference for the FAD-binding domain of the protein. Thus, binding of C12 and D5 near the C-terminal extension of *Xcc*FPR has a deleterious effect on the conformational rearrangement proposed for this region of bacterial FPRs as a requirement to achieve a catalytically competent interaction by stacking the nicotinamide ring of the NADPH hydride donor with the isoalloxazine of the FAD acceptor. Therefore, our docking structural information allows the presence of C12 and D5 not only competing with binding of the pyrophosphate moiety of NADPH in *Xcc*FPR, but also preventing rearrangements at the catalytic site for productive nicotinamide binding.

Collectively, and considering that *Xcc*FPR contributes to bacterial infectivity and the plastidic counterparts have different structural and mechanistic behaviors, this enzyme appears as an interesting potential antimicrobial target to test either individually or in synergy with other targets. Here, we optimized an activity-based HTS that can be used for the discovery of new antibacterial drugs targeting *Xcc*FPR. This method is quick, effective and requires a small quantity of protein. Thus, HTS of two commercial chemical libraries (consisting altogether of 11,120 compounds) was first used to select potential inhibitors of the *Xcc*FPR NADPH-dependent DCPIP-diaphorase activity. For the compounds inhibiting activity over 90% in the HTS assay, we evaluated the effect on *Xcc*FPR in terms of IC_50_, IC_max_ and the percentage of inhibition at IC_max_. The best potential HTS *Xcc*FPR inhibitors were also tested in *Xcc* cells, and one of them was found to have antimicrobial properties. We then searched for compounds sharing structural properties with this one, and determined their effects as *Xcc*FPR inhibitors. Our best performing inhibitors are non-covalent and non-competitive inhibitors containing chloro-phenyl rings fused through a urea-thiadiazol cyclic ring system. Moreover, because they bind to a pocket that structurally and mechanistically differs among bacterial and plastidic FNRs, we were able to find *Xcc*FPR inhibitors with low effects on plastidic enzymes. It is also worth noticing that the length and sequence of the C-terminal extension also allows a subdivision of bacterial FPRs in structural subclasses (Figure 1A) [17,18,19]. Such structural differences among subclasses might also determine the binding or the inhibitory capability of some compounds to particular FPRs, making them subclasses- or species-specific. These would be of great interest for the treatment of infections and for minimizing the selection of resistant bacterial strains. In conclusion, we have developed a method to identify *Xcc*FPR inhibitors and show this enzyme as a promising drug target. Next phase studies, beyond the scope of this work, must focus on the improvement of the potency, selectivity, pharmacokinetics and drug-likeness of these compounds. In addition, for improved inhibitor effects on citrus plants, both healthy and affected by canker, as well as on other bacterial FPRs and cells will have to be tested.

## 3. Materials and Methods

### 3.1. Biological Material and Chemicals

*Xcc*FPR and *An*FNR were produced and purified from *Escherichia coli* cultures as previously reported [11,25]. Samples were prepared in 50 mM Tris/HCl, pH 8.0. Extinction coefficients used to determine concentrations were: ε_450nm_ = 10.7 mM^−1^·cm^−1^ for *Xcc*FPR [26], ε_450nm_ = 9.4 mM^−1^·cm^−1^ for *An*FNR [25], ε_260nm_ = 18.0 mM^−1^·cm^−1^ for NADP^+^ (Sigma-Aldrich, St. Louis, MO, USA) and ε_340nm_ = 6.22 mM^−1^·cm^−1^ for NADPH (Sigma-Aldrich, St. Louis, MO, USA). A library of 10,000 small molecules (Mw < 500) following Lipinski’s rules [27] and dissolved at 4 mM in DMSO was selected from the Maybridge HitFinder Collection (Thermo Fisher Scientific, Geel, Belgium). The chemical library Prestwick (Prestwick Chemical, Illkirch, France), composed of 1120 compounds dissolved at 10 mM in DMSO and with known bioavailability and safety in humans, was also screened. With the purpose of identifying compounds sharing functional groups and geometric arrangement with the HTS C12 hit, a similarity search over the ChEBI and ChemCD databases was conducted. Relevant analogues were selected on the basis of the Tanimoto coefficient > 0.5, the partition coefficient (cLogP), the number of rotatable bonds and the inspection of systems they contained. Selected hits from the libraries and their derivatives were purchased in larger amounts from Molport MolPort, Riga, Latvia), Sigma-Aldrich (Sigma-Aldrich, St. Louis, MO, USA), Prestwick (Prestwick Chemical, Illkirch, France) or Maybridge (Thermo Fisher Scientific, Geel, Belgium), and their extinction coefficients were determined in house and subsequently used to work out their concentrations.

### 3.2. High-Throughput Screening of Chemical Libraries against XccFPR

A massive search for potential inhibitors of the NADPH-dependent DCPIP (Δε_620_^DCPIP^ = 21 mM^−1^·cm^−1^) diaphorase activity of *Xcc*FPR was carried out by HTS (Figure 2) in the presence of 250 μM of each library compound, providing a maximal of 2.5% DMSO to the sample. The relatively high *Xcc*FPR efficiency in the DCPIP activity demanded optimization of the assay to set it in 10 min and in 96-well plates, as well as to ensure optimal detection and to minimize the presence of false positives and negatives. Final reaction solutions were prepared in wells containing 20 nM *Xcc*FPR, 150 μM NADPH and 76 µM DCPIP in a final volume of 100 μL in 50 mM Tris/HCl, pH 8.0. *Xcc*FPR and DCPIP were the last components added to the wells. DCPIP reduction was followed by measuring absorbance changes at 620 nm for 10 min at 25 °C (every well measured every 10 s) using a Synergy^TM^ HT multimode microplate reader (BioTek Instruments, Winooski, VT, USA). Each row had its corresponding negative and positive controls in the absence of both compounds and enzyme (Column 1) and in the absence of compounds but with enzyme (Column 12 in the plate), respectively. Controls also contained 2.5% DMSO. Plate preparations and measurements were carried out row by row to obtain sufficient measuring points at each well for correct evaluation. Plate Columns 2–11 were used for the evaluation of the compounds situated at equivalent positions in the chemical libraries plates. Absorption at 620 nm for each well was plotted against the reaction time and the slopes (observed reaction rates) of the resulting lines in the first 2 min were calculated for every well (Figure 2B). The observed reaction rates for positive controls were taken as those of fully active enzyme. For each well the residual activity (negative control slope) was subtracted. Plate by plate results were integrated in a diagram showing each well as a point producing a reaction rate at certain distance from the positive controls’ average (Figure 2C). Compounds that decreased the reaction rate below the average reaction rate of the controls minus the standard deviation could be preselected as potential inhibitors, but we further reduced the cut-off by selecting only compounds inhibiting more than 90% of the control’s activity. Reaction wells containing precipitates (compound or protein is not soluble under assay conditions) or being colorless (direct reduction of DCPIP by the compound) were discarded. The FAF-Drug4 web server was finally used to identify PAINS among the selected hits [28].

### 3.3. Cell Viability Assays

To evaluate the sensitivity of *Xcc* to C5, C12, C17 and C19, 100 µL of a bacterial suspension (containing ~10^9^ cells mL^−1^) were mixed with 4 mL of SB medium (5 g L^−1^ sucrose, 5 g L^−1^ yeast extract, 5 g L^−1^ peptone, and 1 g L^−1^ glutamic acid, pH 7.0) and 0.7% (*w v*^−1^) molten agar and poured onto SB-1.5% (*w v*^−1^) agar plates supplemented with 25 µg mL^−1^ ampicillin. After hardening, 2 µL of a 10, 100 and 1000 µM solution of each compound were added onto the agar surface. The zones of growth inhibition were photographed after incubation for 24 h at 28 °C. To assess the compound inhibition effect on *Xcc* growth in liquid medium, overnight cultures were subcultured into fresh SB medium at 2% inoculums and grown for 6 h in the presence of 1 mM C12. Samples were removed at 0, 2, 4 and 6 h, serially diluted and plated on SB-1.5% (*w v*^−1^) agar. Colonies were counted after 48 h of incubation at 28 °C.

### 3.4. Analysis of the Effect of Compounds on the Thermal Stability of XccFPR

The increase in fluorescence of the FAD cofactor when dissociated from the protein upon thermal unfolding was used to evaluate *Xcc*FPR stability under different conditions and to assess related midpoint temperature for flavin release (T_mFAD_) [29,30]. Fluorescence thermal denaturation curves were monitored on 96-well plates using a Stratagene Mx3005P qPCR detection system (Agilent Technologies Inc., Santa Clara, CA, USA). A FAM filter set (λ_exc_ = 492 nm; λ_em_ = 516 nm), which overlaps the fluorescence spectrum of the isoalloxazine ring was used to evaluate *Xcc*FPR midpoint temperature for flavin release (T_mFAD_) when free or in the presence of selected compounds. Measurements were performed using 4 µM protein in 50 mM Tris/HCl, pH 8.0 in a 100 µL final volume. Each measurement was done in triplicate.

### 3.5. Steady-State Kinetic Parameters of XccFPR and Inhibitory Effect of Selected Hits

When assayed in regular cuvettes, the DCPIP diaphorase activity of *Xcc*FPR was determined at 25 °C using a Cary 100 (Agilent Technologies Inc., Santa Clara, CA, USA) spectrophotometer as previously established [25]. Final reaction mixtures contained 20 nM *Xcc*FPR, 95 µM DCPIP and NADPH in the 0–100 μM range in 50 mM Tris/HCl, pH 8.0 at 25 °C. To determine the IC_50_, IC_max_ and *Xcc*FPR residual activity of the hits, the DCPIP diaphorase activity was assayed at different concentrations of each potential inhibitor (0–800 µM range) with 50 µM NADPH. Positive controls (without any hit compound) were included in every reaction set. Experiments designed to assess the type of inhibition and the inhibition constant (*K_i_*) of high-performance inhibitors were carried out varying both NADPH and inhibitor concentrations in the 0–50 µM and 0–100 μM ranges, respectively. The DMSO concentration was kept at 2% in all samples. All kinetic experiments were performed in triplicate. The effect of the inhibitors on *K_m_* and *k_cat_* was determined by fitting the datasets to the Michaelis-Menten model. Additionally, data were globally fit to either Lineweaver-Burk equations for uncompetitive or non-competitive inhibition, yielding *K_i_* for each compound (Equations (1) and (2), respectively).

(1) $$\frac{\left[e\right]}{V_{0}}=\frac{\left(1+\frac{\left[I\right]}{{K^{\prime }}_{i}}\right)}{k_{cat}}+\frac{K_{m}}{k_{cat}}\cdot \frac{1}{\left[S\right]}$$

(2) $$\frac{\left[e\right]}{V_{0}}=\frac{\left(1+\frac{\left[I\right]}{K_{i}}\right)}{k_{cat}}+\frac{\left(1+\frac{\left[I\right]}{K_{i}}\right)\cdot K_{m}}{k_{cat}}\cdot \frac{1}{\left[S\right]}$$

Measurements were done in triplicate and errors in determined *k_cat_*, *K_m_* and *K_i_* were in general within ±10, ±10 and ±15% of the value, respectively.

### 3.6. Docking of Compounds C12 and D5 to XccFPR

The GOLD 5.5 software [31] (using the GoldSore scoring function, 15 genetic algorithm runs for each ligand and default parameters) and the crystal coordinates of *Xcc*FPR (PDB ID: 4b4d) [18] (after removal of ions and water molecules) were used to obtain the interaction models with C12 and D5. The structure was described with the charmm36 force field using CHARMM c39b1 [32,33] and protonated using PROPKA [34]. C12 and D5 geometries were optimized using the functional B3LYP/def2-SVP+GD3BJ [35] and a water-like polarizable continuum model using the Gaussian09 rev.D01 software [36]. FAD and ligand parameters were generated using CGenFF [37]. An initial docking calculation using the whole protein as the target was done to identify the most probable C12 and D5 docking sites on *Xcc*FPR. For subsequent refined docking runs, the binding site was constructed in the readily identified cavity that lies between G115, T182 and N224, and flexibility was enabled for R183, R191 and S222 using rotamer libraries over 15 genetic algorithm runs. Short molecular dynamics (MD) simulations, using CHARMM and periodic boundary conditions with explicit TIP3P solvent, were carried out to improve protein-ligand coupling. The receptor-ligand complexes were minimized over 100 ps, and equilibrated for 100 ps in NVT conditions. Production runs were performed for an additional 100 ps. Neutralization was performed with a Monte-Carlo scheme. The time step was set at 1 fs for all simulations. Ligand-protein interaction energies were calculated over the production runs and the main interaction sites were analyzed.

### 3.7. Data Analysis and Statistics

Structural alignments were carried out using the PROMALS3D server [38] and the structures of the bacterial FPRs from *Xcc* (PDB 4b4d; Subclass 1A), *Azotobacter vinelandii* (PDB 1a8p; Subclass 1A), *Rhodobacter capsulatus* (PDB 2bgi; Subclass IB) and *E. coli* (PDB 1fdr; Class II) and the plastidic FNRs from *Anabaena* PCC 7119 (PDB 1que) and *Pisum sativum* (PDB 1qg0). Origin (OriginLab) was used for data representation and fitting. Results are expressed as the mean ± the standard deviation (SD). When indicated, one-way analysis of variance (ANOVA) was performed to determine statistical significance. Chemical structures were drawn using ChemDraw Professional 16 (PerkinElmer Informatics). PyMol [39] and UCS-Chimera [40] were used to produce structural figures.

## Figures and Tables

**Table 1:** Effect of HTS hits on the NADPH-dependent DCPIP diaphorase activity of *Xcc*FPR. Kinetic measurements were carried out at 25 °C, in 50 mM Tris/HCl, 2% DMSO, pH 8.0, using 20 nM *Xcc*FPR, with saturating DCPIP (95 µM) and NADPH (50 μM), and varying the hits concentration in the 0–800 μM range (*n* > 2, means ± SD). IC_50_ and IC_max_ stand for the concentration of the compound causing 50% enzyme inhibition and maximal inhibition, respectively. All shown *Xcc*FPR inhibition percentages at IC_max_ are statistically significant (***, *p* < 0.0001).

HTS Hit	IC_50_ (µM)	IC_max_ (µM)	% Inhibition at IC_max_	HTS Hit	IC_50_ (µM)	IC_max_ (µM)	% Inhibition at IC_max_
1	110 ± 1	130 ± 1	84 ± 4	19	17 ± 1	80 ± 1	100 ± 1
2	27 ± 1	300 ± 1	95 ± 3	20	53 ± 2	100 ± 1	100 ± 1
3	190 ± 1	300 ± 3	31 ± 8	21	14 ± 1	80 ± 1	62 ± 7
4	36 ± 1	80 ± 1	86 ± 1	22	58 ± 2	150 ± 1	100 ± 4
5	46 ± 1	150 ± 1	98 ± 1	23	>604	>1000	73 ± 3
6	>605	>1000	~70	24	35 ± 1	120 ± 1	43 ± 4
7	85 ± 16	450 ± 1	97 ± 7	25	91 ± 1	>500	~91
8	79 ± 1	250 ± 1	88 ± 9	26	33 ± 2	120 ± 1	94 ± 1
9	147 ± 1	250 ± 1	60 ± 4	27	89 ± 1	120 ± 1	92 ± 3
10	98 ± 1	150 ± 1	57 ± 7	28	>652	>1500	81 ± 2
11	78 ± 1	140 ± 1	92 ± 5	29	11 ± 2	50 ± 1	27 ± 2
12	7.7 ± 1.0	45 ± 1	100 ± 2	30	29 ± 1	80 ± 1	92 ± 2
13	95 ± 1	180 ± 1	80 ± 3	31	90 ± 1	150 ± 3	85 ± 1
14	127 ± 1	200 ± 1	85 ± 4	32	>2720	>4000	~56
15	30 ± 1	150 ± 1	93 ± 4	33	>1920	>2800	~53
16	58 ± 1	150 ± 1	79 ± 6	34	210 ± 1	>1500	~87
17	27 ± 2	150 ± 1	91 ± 4	35	63 ± 1	100 ± 2	100 ± 1
18	24 ± 1	150 ± 1	100 ± 1				

**Table 2:** Effect of C12 derived hits on the NADPH-dependent DCPIP diaphorase activity of *Xcc*FPR. Kinetic measurements carried out at 25 °C, in 50 mM Tris/HCl, 2% DMSO, pH 8.0, using 20 nM *Xcc*FPR, with saturating DCPIP (95 μM) and NADPH (50 μM), and varying the compounds concentration in the 0–800 μM range (*n* > 2, means ± SD). IC_50_ and IC_max_ stand for the concentration of the compound causing 50% enzyme inhibition and maximal inhibition, respectively. All shown *Xcc*FPR inhibition percentages at IC_max_ are statistically significant (***, *p* < 0.0001).

C12 Derived Compound	IC_50_ (µM)	IC_max_ (µM)	% Inhibition at IC_max_
D1	76 ± 1	200 ± 1	98 ± 1
D2	26 ± 1	175 ± 1	100 ± 1
D3	>5000	-	-
D4	113 ± 1	400 ± 1	78 ± 2
D5	6.6 ± 1.1	23 ± 2	100 ± 2
D6	>1500	-	-
D7	151 ± 1	220 ± 1	84 ± 1
D8	38 ± 1	150 ± 1	100 ± 1

**Table 3:** Effect of *Xcc*FPR inhibitors on the NADPH-dependent DCPIP diaphorase activity of *An*FNR. Kinetic measurements carried out at 25 °C, in 50 mM Tris/HCl, 2% DMSO, pH 8.0, using 4 nM *An*FNR, with saturating DCPIP (95 µM) and NADPH (50 μM), and varying the concentration of the compounds in the 0–800 μM range (*n* = 3, means ± SD). IC_50_ and IC_max_ stand for the concentration of the compound causing 50% enzyme inhibition and maximal inhibition, respectively.

Inhibitors	IC_50_ (µM)	IC_max_ (µM)	% Inhibition at IC_max_
D2	27 ± 1	100 ± 2	99 ± 11
D5	102 ± 1	>300	~47
D8	122 ± 1	>350	~73

**Table 4:** Inhibition constants and mechanisms for the best-performing inhibitors of the diaphorase activity of *Xcc*FPR. Values calculated by globally fitting the experimental data to the corresponding Lineweaver–Burk inhibition model. Measurements were carried out at 25 °C, in 50 mM Tris/HCl, 2% DMSO, pH 8.0 (*n* = 3, means ± SD).

	*K_i_* (μM)	Inhibition Mechanism
C12	4.2 ± 0.9	Non-competitive
D2	28 ± 2	Uncompetitive
D5	4.3 ± 1.1	Non-competitive
D8	62 ± 19	Non-competitive

## Supplementary Materials

Additional supporting information may be found in the online version of this article at the publisher’s website. It contains; Table S1 showing the midpoint temperature values for *Xcc*FPR FAD cofactor release (T_mFAD_), Table S2 with the properties of HTS hits of the NADPH-dependent DCPIP diaphorase activity of *Xcc*FPR, Table S3 with the properties of C12 related hits of the NADPH-dependent DCPIP diaphorase activity of *Xcc*FPR, Figure S1 containing the Michaelis-Menten representation of the DCPIP dependent diaphorase activity of *Xcc*FPR, Figure S2 showing the workflow to identify additional compounds outside the HTS libraries, Figure S3 showing the results from the docking analysis of the best five poses of the inhibitors C12 and D5, and Figure S4 containing MD trajectories.
