# Supplementary material for: Identification of Inhibitors Targeting Ferredoxin-NADP+ Reductase from the Xanthomonas citri subsp. citri Phytopathogenic Bacteria
Source: Molecules. 2017 Dec 24;23(1):29. doi: 10.3390/molecules23010029 (PMC5943930; doi:10.3390/molecules23010029)
Supplement: Supplementary file 1 [file molecules-23-00029-s001.docx]

**Supplementary Material**

**Identification of inhibitors targeting the Ferredoxin-NADP^+^ reductase from the *Xanthomonas citri* subsp *citri* phytopathogenic bacteria**

**Marta Martínez-Júlvez^1^, Guillermina Goñi^1^, Daniel Pérez-Amigot^1^, Rubén Laplaza^1,2^, Irina Ionescu^1,a^, Silvana Petrocelli^3^, María Laura Tondo^3^, Javier Sancho^1^, Elena Orellano^3^, and Milagros Medina^1,*^**

^1^ Departamento de Bioquímica y Biología Molecular y Celular, Facultad de Ciencias, and Institute of Biocomputation and Physics of Complex Systems (BIFI-IQFR and CBsC-CSIC Joint Units), Universidad de Zaragoza, Spain

^2^ Departamento de Química Física, Universidad de Santiago de Compostela, Spain

^3^ Molecular Biology Division, Instituto de Biología Molecular y Celular de Rosario (IBR), CONICET, Facultad de Ciencias Bioquímicas y Farmacéuticas, Universidad Nacional de Rosario, Argentina.

Supporting contains; Table S1 showing the midpoint temperature values for *Xcc*FPR FAD cofactor release (T_mFAD_), Table S2 with properties of HTS hits of the NADPH-dependent DCPIP diaphorase activity of *Xcc*FPR, Table S3 with properties of C12 related hits of the NADPH-dependent DCPIP diaphorase activity of *Xcc*FPR, Figure S1 contains Michaelis-Menten representation of the DCPIP dependent diaphorase activity of *Xcc*FPR, Figure S2 shows the work flow to identify additional compounds outside the HTS libraries, Figure S3 shows results from the docking analysis of the best five poses of the inhibitors C12 and D5, and Figure S4 contains MD trajectories.

**SUPPORTING TABLES**

**Table S1. Midpoint temperature values for *Xcc*FPR FAD cofactor release (T_mFAD_).** Values obtained by fluorescence thermal denaturation curves. Measurements were carried out at 25 °C, in 50 mM Tris/HCl. Protein concentrations was 4 µM.

| Substances in  Sample | T_mFAD_ (°C) | ΔT_mFAD_ (°C) |
| --- | --- | --- |
| --- | 41.0 ± 0.1 | - |
| DMSO (2 %) | 40.6 ± 0.1 | -0.4±0.2 |
| DMSO (5 %) | 39.0 ± 0.1 | -1.8±0.2 |
| DMSO (10 %) | 37.7 ± 0.1 | -3.1±0.2 |
| NADP^+^ (50 µM) | 47.2 ± 0.1 | 6.2±0.2 |
| NADP^+^ (50 µM), DMSO (2 %) | 46.7 ± 0.1 | 5.7±0.2 |
| D2 (2 mM), DMSO (2 %) | 41.7 ± 0.1 | 0.7±0.2 |
| D2 (2 mM), NADP^+^ (50 µM), DMSO (2 %) | 46.0 ± 0.1 | 5.0±0.2 |
| D5 (1 mM), DMSO (2 %) | 41.4 ± 0.1 | 0.4±0.2 |
| D5 (1 mM), NADP^+^ (50 µM), DMSO (2 %) | 46.9 ± 0.1 | 5.9±0.2 |
| D10 (2 mM), DMSO (2 %) | 41.0 ± 0.1 | 0 |
| D10 (2 mM), NADP^+^ (50 µM), DMSO (2 %) | 46.0 ± 0.2 | 5.0±0.3 |

**Table S2. HTS hits of the NADPH-dependent DCPIP diaphorase activity of *Xcc*FPR**

| **HIT** | **IUPAC name** | **structure** | **cLogP** | **Mw** |
| --- | --- | --- | --- | --- |
| C1 | Benzethonium chloride | 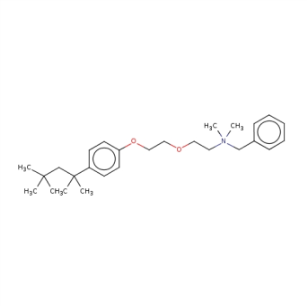 | 4 | 448.09 |
| C2 | 2-Phenyl-1,2-benzoselenazol-3-one(ebselen) | 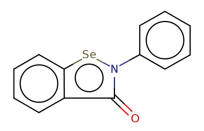 | 2.048 | 274.18 |
| C3 | benzalkonium chloride | 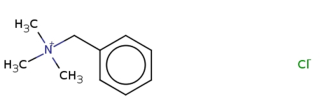 | -1.103 | 185.7 |
| C4 | 1,3-dichloro-2-(4-nitrophenoxy)benzene | 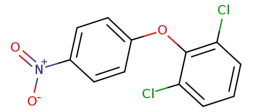 | 4.694 | 284.09 |
| C5 | N-[3,5-bis(trifluoromethyl)phenyl]-2-[(5-chloro-1,3-benzoxazol-  2-yl)sulfanyl]acetamide | 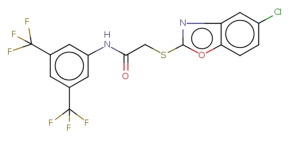 | 6.323 | 454.77 |
| C6 | 4-(2-fluorophenyl)-N,N-bis(propan-  2-yl)benzamide | 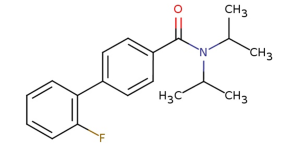 | 4.752 | 299.39 |
| C7 | 3-(1-ethynylcyclohexyl)-1-(6-{[(1-ethynylcyclohexyl)  carbamoyl]amino}hexyl)  urea | 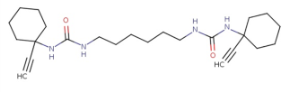 | 3.817 | 414.60 |
| C8 | 2-(benzylsulfanyl)-5-chloro-1,3-benzothiazole | 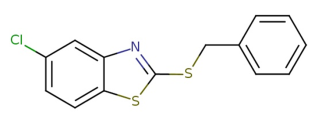 | 5.242 | 291.81 |
| C9 | 5-methyl-4-phenyl-N-({[3-(trifluoromethyl)phenyl]  carbamoyl}amino)-1,3-oxazole-2-carboxamide | 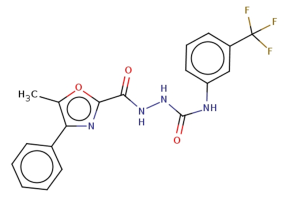 | 4.990 | 404.35 |
| C10 | 4-amino-3,5-dichloro-N-(2,4,5-trichlorophenyl)benzamide | 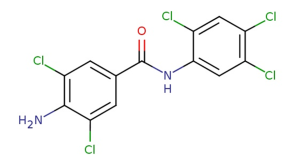 | 5.788 | 384.46 |
| C11 | 4-(1,3-dithiolan-2-yl)-2-methoxy-6-nitrophenyl 2,5-dichlorobenzene-1-sulfonate | 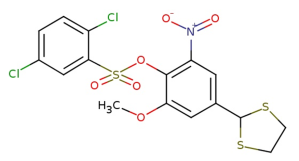 | 5.157 | 482.36 |
| C12 | 1-(3-chloro-4-fluorophenyl)-3-[3-(4-chlorophenyl)-  4-cyano-  5-(methylsulfanyl)thiophen-2-yl]urea | 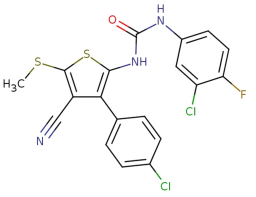 | 4.781 | 452.34 |
| C13 | 2-(benzylamino)-2-(3,4-dichlorophenyl)acetonitrile | 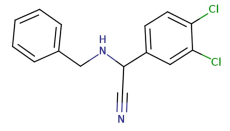 | 4.739 | 291.18 |
| C14 | ethyl 5-benzamido-4-cyano-3-methyl-1-phenyl-1H-pyrrole-2-carboxylate | 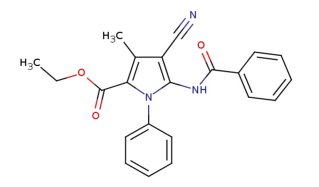 | 4.16 | 373.41 |
| C15 | 3-{3-[(2,4-dichlorophenyl)sulfanyl]-3-oxopropyl}-2,3-dihydro-1,3-benzoxazol-2-one | 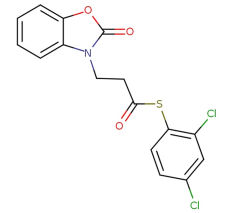 | 4.61 | 368.23 |
| C16 | 1-{3-[({[(4Z)-3,4-dihydro-1H-2-benzothiopyran-4-ylidene]amino}oxy)methyl]-4-methoxyphenyl}ethan-1-one | 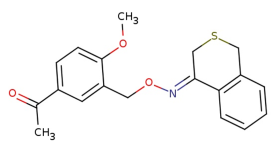 | 4.066 | 341.43 |
| C17 | 4-bromo-3-[(1E)-[2-(2,4-dichlorophenyl)hydrazin-  1-ylidene]methyl]-1-methyl-1H-pyrazole | 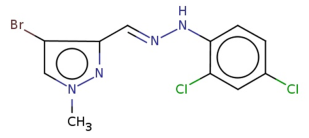 | 3.935 | 348.03 |
| C18 | 2-({2-[(2-aminophenyl)sulfanyl]-6-nitro-4-(trifluoromethyl)phenyl}  sulfanyl)aniline | 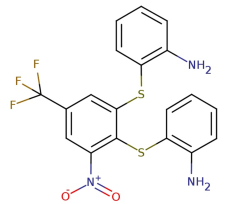 | 6.08 | 437.46 |
| C19 | N-(2-{[3-cyano-6-methyl-5-(2-methyl-1,3-thiazol-4-yl)pyridin-2-yl]sulfanyl}ethyl)-3-(trifluoromethyl)benzene-1-sulfonamide | 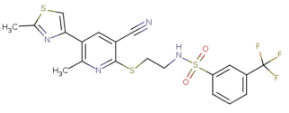 | 4.783 | 498.56 |
| C20 | N-(2H-1,3-benzodioxol-5-yl)-4,6-dimethyl-1H-indole-  2-carboxamide | 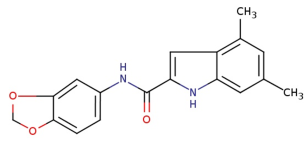 | 3.766 | 308.34 |
| C21 | 3,5-diethyl 4-{5-[2-chloro-5-(trifluoromethyl)phenyl]  furan-2-yl}-2,6-dimethyl-1,4-dihydropyridine-3,5-dicarboxylate | 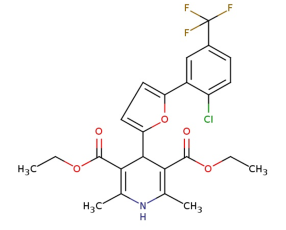 | 5.98 | 497.9 |
| C22 | (2E,6E)-2,6-bis[(thiophen-2-yl)methylidene]cyclohexan-1-one | 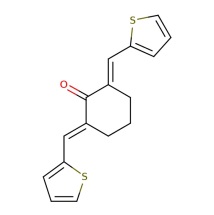 | 5.03 | 286.41 |
| C23 | 2-[4-(4-chlorophenyl)-2H-1,3-dithiol-  2-ylidene]propanedinitrile | 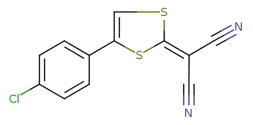 | 4.337 | 276.76 |
| C24 | 4-cyclohexyl-2-{[(2,5-dimethylphenyl)methyl]  sulfanyl}-6-oxo-1,6-dihydropyrimidine-5-carbonitrile | 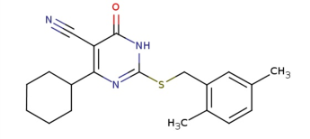 | 5.011 | 353.48 |
| C25 | (E)-({[5-(5-bromothiophen-2-yl)-1,3-thiazol-2-yl]amino}methylidene)amino 4-nitrobenzoate | 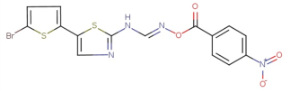 | 4.755 | 453.29 |
| C26 | methyl 3-[2-(thiophen-2-yl)acetamido]thiophene-2-carboxylate | 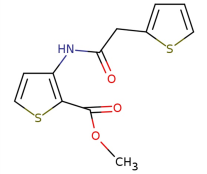 | 2.778 | 281.34 |
| C27 | 3-amino-5-[(cyanomethyl)sulfanyl]-4-(propane-2-sulfonyl)thiophene-2-carbonitrile | 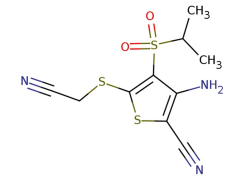 | 2 | 301.4 |
| C28 | N-(4-butyl-2-methylphenyl)-2-[(5-cyano-4-cyclohexyl-6-oxo-1,6-dihydropyrimidin-2-yl)sulfanyl]acetamide | 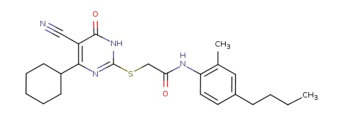 | 5.071 | 438.59 |
| C29 | N-{2-[3-(4-chlorophenyl)-1,2,4-oxadiazol-5-yl]phenyl}-2,2,2-trifluoroacetamide | 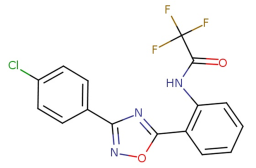 | 4.556 | 367.71 |
| C30 | 2-chloro-6H-5-thia-7-azatetraphene-12-carboxylic acid | 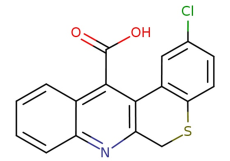 | 4.859 | 327.78 |
| C31 | 2-(4-bromothiophen-2-yl)-1,3-dithiolane | 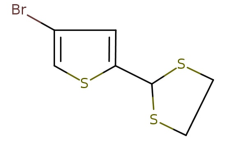 | 3.989 | 267.22 |
| C32 | 3-(5-methyl-2-oxo-2,3-dihydro-1,3-benzoxazol-3-yl)-N'-[3-(thiophen-2-yl)-1,2,4-oxadiazole-5-carbonyl]propanehydrazide | 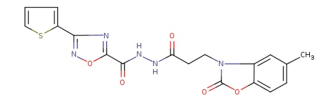 | 1.866 | 325.36 |
| C33 | ethyl 2-[3-(3,4,5-trimethoxyphenyl)  propanamido]acetate | 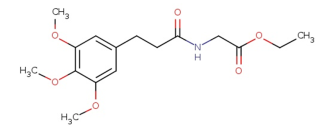 | 1.324 | 325.36 |
| C34 | 2-amino-4-(4-chlorophenyl)-9-fluoro-4H,5H-pyrano[3,2-c]chromene-3-carbonitrile | 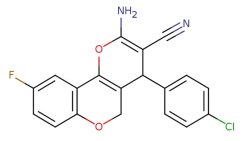 | 4.09 | 354.77 |
| C35 | 2-oxo-2-phenylethyl 3-iodobenzoate | 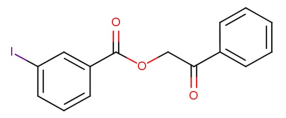 | 3.331 | 366.15 |

**Table S3. C12 related hits of the NADPH-dependent DCPIP diaphorase activity of *Xcc*FPR.**

| C12 related  hits | IUPAC  name | Formula | clogP | Mw |
| --- | --- | --- | --- | --- |
| D1 | 3-(3-chlorophenyl)-1-[2-(thiophene-2-sulfonamido)phenyl]urea | 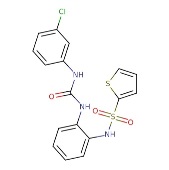 | 4.846 | 407.89 |
| D2 | N-[4-cyano-5-(methylthio)-2-thienyl]-N'-[3-trifluoromethyl)phenyl]urea | 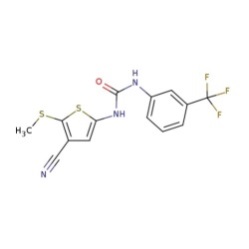 | 5.004 | 357.37 |
| D3 | 1-(3-Chloro-4-fluoro-phenyl)-3-(3-chloro-phenyl)-urea | 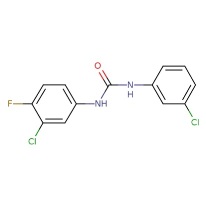 | 4.777 | 299.13 |
| D4 | N-[3-(4-chlorophenyl)-4-cyano-5-(methylsulfanyl)thiophen-2-yl]morpholine-4-carboxamide | 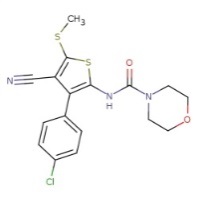 | 4.526 | 393.90 |
| D5 | 1-[5-(4-Chloro-benzylsulfanyl)-[1,3,4]thiadiazol-2-yl]-3-(4-chloro-phenyl)-urea | 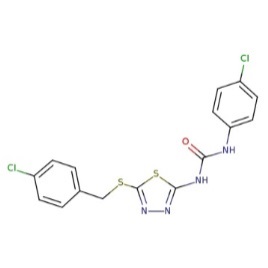 | 5.781 | 411.32 |
| D6 | 3-(3-chlorophenyl)-4-cyano-5-(methylsulfanyl)thiophene-2-carboxylic acid | 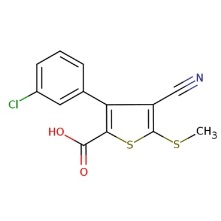 | 4.36 | 309.78 |
| D7 | 1-(2,6-dimethylphenyl)-3-thiophen-  2-ylurea | 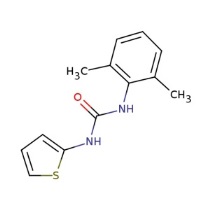 | 4.009 | 246.33 |
| D8 | 4-(4-chlorobenzenesulfonyl)-N-(2,4-difluorophenyl)-3-methylthiophene-2-carboxamide | 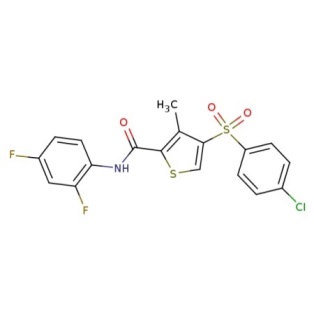 | 5.073 | 427.86 |

**Supplementary Figures**

**Figure S1**. Michaelis-Menten plot of the *Xcc*FPR NADPH-dependent DCPIP diaphorase activity. Reaction rates were measured in 50 mM Tris/HCl pH 8.0 containing 20 nM *Xcc*FPR, 100 µM DCPIP and 0-100 μM NADPH at 25 °C (n=3, means ± SD).

**
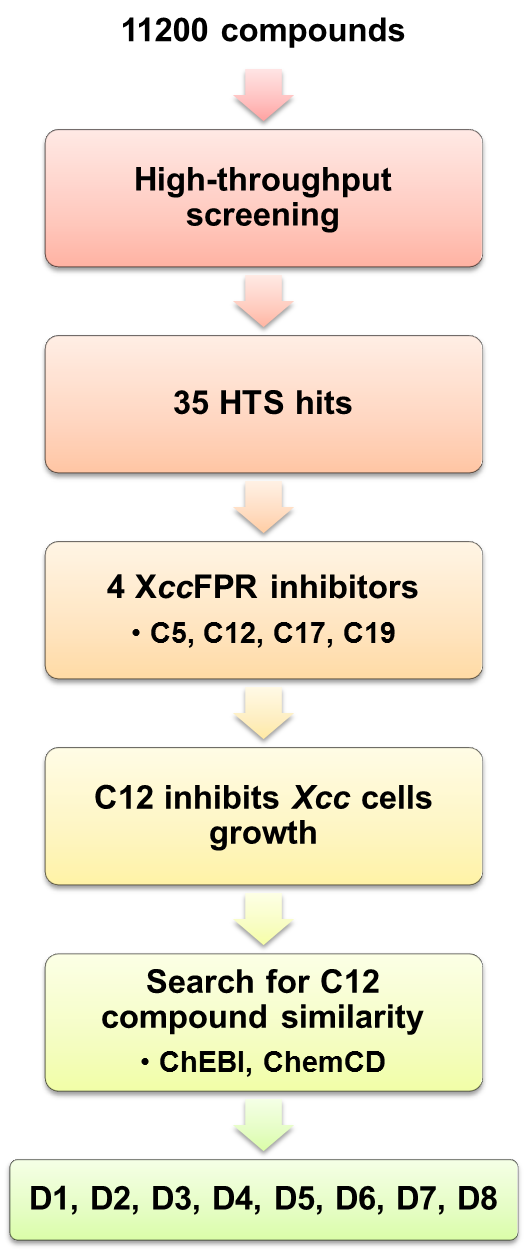
**

**Figure S2**. Workflow diagram to identify compounds related to the best performing inhibitor identified by HTS.

**
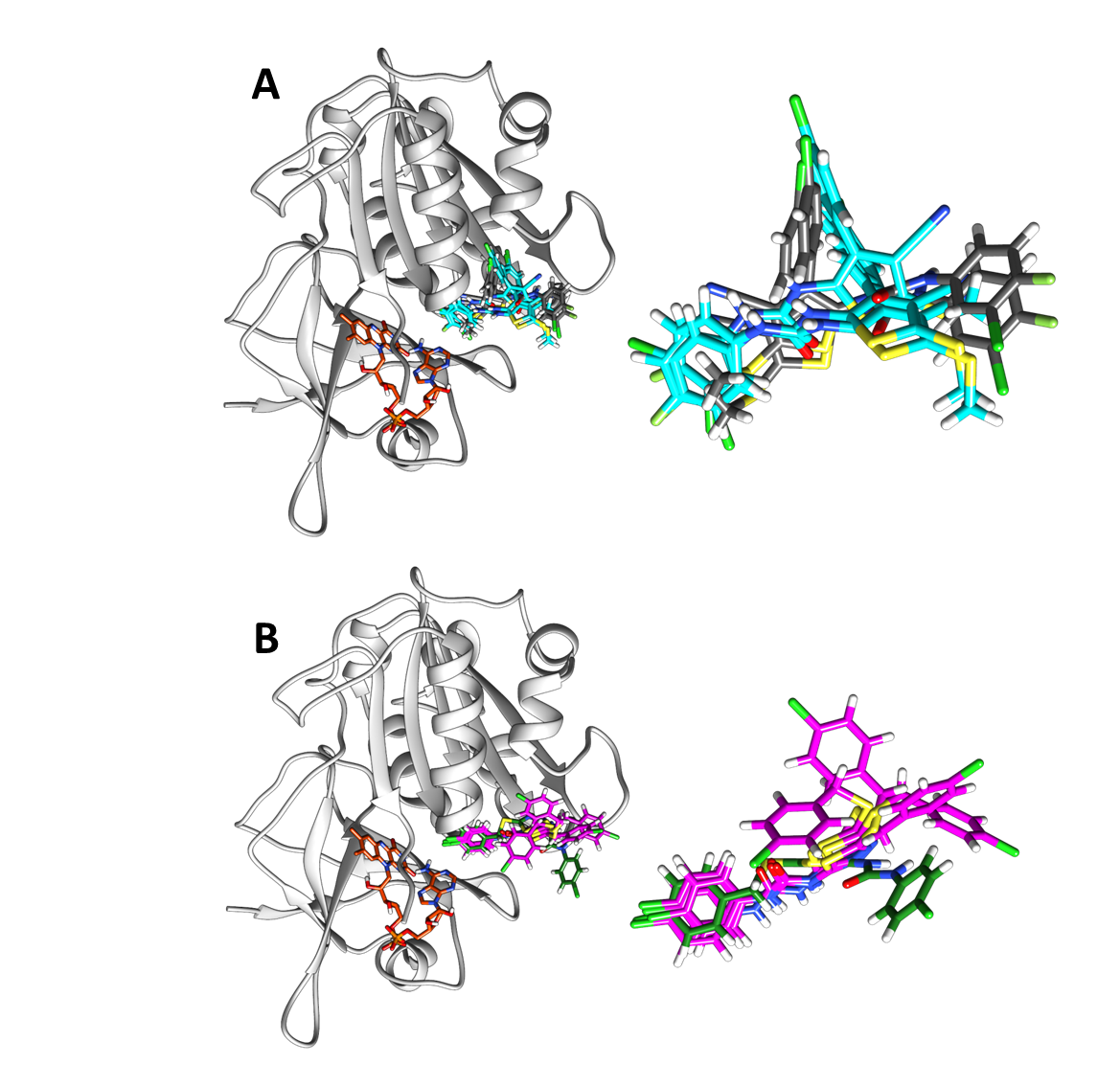
**

**Figure S2**. Docking analysis of the best five poses of the inhibitors (A) C12 and (B) D5 of *Xcc*FPR. Protein is shown as grey cartoon, FAD as sticks with carbons in orange and inhibitors are in sticks with carbons colored by cluster. Top ranked clusters are shown with carbons in cyan (A) and magenta (B). Panels on the right represent the spatial distribution of the aforementioned poses in detail to underline the conservation patterns.

**
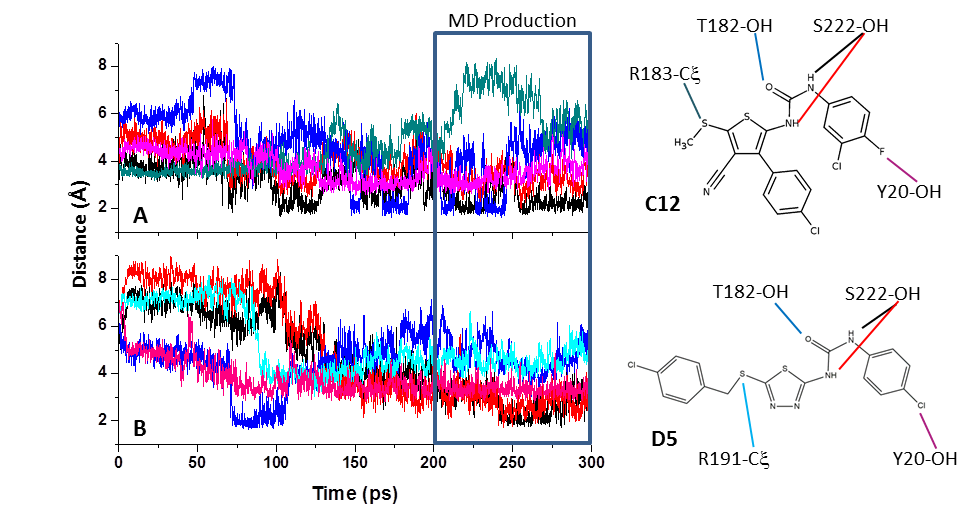
**

**Figure S3**. Trajectories for some interactions of *Xcc*FPR with (A) C12 and (B) D5 along the MD simulation experiment. Initial interacting complexes correspond to the best docking pose for each inhibitor. Charts on the right indicate by color the specific atom-to-atom distance for which time evolution is drawn on the left panels. The box indicates the time frame corresponding to the MD production.
